# The Association Between Price and Household Purchasing of No-Alcohol Beer in Great Britain and Spain

**DOI:** 10.3390/nu18203310

**Published:** 2026-10-09

**Authors:** Brayden Anderson Jané, Alejandro Sonis, Jürgen Rehm, Peter Anderson

**Affiliations:** 1 Cambridge School Online, Camboro Business Park, Girton, Cambridge CB3 0QH, UK; 2 Department of Health Economics, Ministry of Health, Av. 9 de Julio 1925, Cdad. Autónoma de Buenos Aires C1072, Argentina; alejandrosonis@yahoo.com.ar; 3 Institute for Mental Health Policy Research, Centre for Addiction and Mental Health, Toronto, ON M5T 1R8, Canada; jtrehm@gmail.com; 4 Dalla Lana School of Public Health, University of Toronto, Toronto, ON M5T 3M7, Canada; 5 Department of Psychiatry, University of Toronto, Toronto, ON M5T 1R8, Canada; 6 Institute of Medical Science, University of Toronto, Toronto, ON M5S 3H2, Canada; 7 Centre for Interdisciplinary Addiction Research, University Medical Center Hamburg-Eppendorf, 20246 Hamburg, Germany; 8 Program on Substance Abuse & WHO European Region Collaboration Centre, Public Health Agency of Catalonia, 08005 Barcelona, Spain; 9 Independent Researcher, Cambridge CB23 6LN, UK; peteranderson.mail@gmail.com

**Keywords:** no-alcohol beer, price, household purchases, Great Britain, Spain, Kantar data

## Abstract

**Background:** The ethanol in alcoholic beverages is toxic to human health. Reducing alcohol-related harm, a major public health priority, requires reductions in the amount of ethanol consumed. Alongside policies that increase alcohol prices and restrict availability, encouraging substitution from regular-strength beer to no-alcohol beer may reduce population-level alcohol consumption. Pricing policies could encourage substitution and thus reduce ethanol consumption and harm to human health. **Methods:** We analyze longitudinal household purchase data from Kantar Worldpanel for Great Britain (2015–2020) and Spain (2017–2022), made up of over six million alcohol purchases. We examine the relationships between the price and purchases of no-alcohol beer. **Results:** Among 85,690 households analyzed, 7509 (8.8%) newly purchased no-alcohol beer. A 10-percentage-point decrease in price relative to the sample price range for no-alcohol beer (ABV = 0.0%) was associated with a 157% increase in the odds of newly purchasing no-alcohol beer, representing an absolute increase of 11.1 percentage points (from 8.8% to 19.9%) in the proportion of households newly purchasing no-alcohol beer. A 10-percentage-point increase in price relative to the sample range for regular-strength beer (ABV > 3.5%) was associated with an 11.3% increase in the odds of households newly purchasing no-alcohol beer, representing an absolute increase of 0.9 percentage points (from 8.8% to 9.7%). **Conclusions:** These observed purchasing patterns suggest that pricing strategies—such as lowering the relative price of no-alcohol beers alongside increases for regular-strength beers, could align with consumer shifts toward lower-alcohol alternatives.

## 1. Introduction

The alcohol in alcoholic beverages is toxic to many bodily systems, is genotoxic and is a carcinogen, being a cause of cancers of the oral cavity, pharynx, larynx, esophagus, colorectum, liver (hepatocellular carcinoma) and female breast [1]. Within the European Union, a population of some 450 million people, alcohol is responsible for a little under 300,000 deaths a year, with cancer the top cause of alcohol-related deaths (29% of all deaths due to alcohol), followed by liver cirrhosis (20% of all deaths due to alcohol), and cardiovascular diseases (19% of all deaths due to alcohol) [2]. Globally, alcohol is the annual cause of some 2.6 million deaths [3].

The most effective way to minimize the harm done by alcohol is to reduce its consumption [3]. People can be motivated to drink less alcohol by making alcohol more expensive, decreasing its availability, and restricting its advertising [3,4]. An additional measure would be to encourage people to switch drinking to lower-alcohol-strength products, including no-alcohol beers [5,6]. In its 2022–2030 action plan, WHO called on alcohol producers to “substitute, whenever possible, higher-alcohol products with no-alcohol and lower-alcohol products in their overall product portfolios, with the goal of decreasing the overall levels of alcohol consumption in populations and consumer groups” [7]. A key policy that favours the substitution of higher strength alcohol products with lower strength products is an alcohol tax based on the dose of alcohol across all products [8].

European consumers are increasingly buying and drinking lower-strength alcohol products over time, with some two fifths doing so to drink less alcohol [8,9]. It tends to be younger, more socially advantaged men, and existing heavier buyers and drinkers of alcohol, who take up lower-strength alcohol products.

Previous evidence from Great Britain [10] and Spain [11] indicate that households that newly buy no-alcohol beer substitute purchases of regular-strength beer, reducing overall purchases of grams of alcohol. Understanding how the prices of no-alcohol beer and regular-strength beer influence household purchasing behaviour could help design effective pricing policies and health interventions that in turn diminish the harm done by alcohol [8].

In this paper, we focus on no-alcohol beer, as this is the beverage category that is most developed and most commonly purchased with respect to no-alcohol products [8].

A previous study using British data, predominantly for the years 2015–2018, studied the potential impact of prices and price promotions on purchases of beer [12]. The study found that, compared to regular-strength beer, no-alcohol beer was less frequently on price promotion, was cheaper per litre, and saw similar volume increases from lower prices regardless of whether on price promotion, whereas regular-strength beer was more often on price promotion, cost more per litre, and experienced a higher purchase volume when a lower price was tied to a price promotion. Other than this similar study, we are not aware of any studies that have investigated the specific relationship between the prices of no-alcohol beer and regular-strength beer with the first recorded purchase and purchased volume of no-alcohol beer in the British and Spanish markets.

Using household-level alcohol purchasing data from Great Britain and Spain, we analyze how changes in the prices of no-alcohol beer and regular-strength beer are associated with: (1) the odds that a household newly purchases no-alcohol beer; and (2), the volume of no-alcohol beer purchased. Based on price sensitivity evidence [13], we hypothesize: lower prices of no-alcohol beer will be associated with increases in both the odds of first-time purchase and the volume of no-alcohol beer purchased; and higher prices of regular-strength beer will be associated with increases in the odds of households buying no-alcohol beer, but with a smaller effect on the volume of no-alcohol beer purchased. Further, based on the published literature [8], we hypothesize that a range of household characteristics will moderate these associations.

## 2. Methods

### 2.1. Study Design

We use regression analyses (SPSS v31.0 procedure GENLIN [14]) to explore the potential associations between prices of both no-alcohol (ABV = 0.0%) and regular-strength (ABV > 3.5%) beer, and the odds of newly purchasing and the volumes of purchases of no-alcohol beer. We use an ABV of 3.5% as a commonly accepted cut-off point between low-alcohol and regular-strength beers [8].

### 2.2. Data Source

Our data sources were Kantar Worldpanel’s (KWP) household shopping panels. In Great Britain, KWP comprises approximately 30,000 British households at any one time, recruited via stratified sampling, with targets set for region, household size, age of main shopper and occupational group, with the panel being representative of households in Great Britain as a whole (see [15]). In Spain, KWP comprises approximately 12,000 Spanish households at any one time, recruited via stratified sampling, with targets set for the province, household size, and age of the main shopper; the panel is representative of households in Spain as a whole (see [16]). In both panels, households provide demographic information when joining the panel, followed by annual updates and quality checks. Using barcode scanners, households record all alcohol purchases brought into the home from all store types, including Internet shopping.

We obtained data pre-prepared from KWP panels on take-home purchasing of alcohol products (including no-alcohol beer) in Great Britain for the years 2015–2020 [15] and in Spain for the period from the second quarter of 2017 to the end of the first quarter of 2022 [16].

Data were available on the category of product purchased (beer, wine, spirits, etc.), volume and alcohol by volume (ABV) of product purchased, and price paid of product purchased for all alcohol purchases for all households and for every day of purchase of an alcohol product (including no-alcohol beer products) that the household was in the panel. As we were particularly interested in households that, in general, regularly bought alcohol (as these are the households where a switch from regular-strength beer to no-alcohol beer may have a greater impact in reducing overall alcohol purchases), we excluded from the analysis those households that throughout their time in the panel made an alcohol purchase less than once every three months (infrequent buyers of alcohol).

With respect to the data sources, it should be noted that whilst Kantar Worldpanel provided the original raw data free of charge (see refs. [10,11]), Kantar Worldpanel received reimbursement from AB InBev to cover the costs of the British data and from Heineken to cover the costs of the Spanish data, with neither the company nor Kantar Worldpanel having any communication or role in the study design, data analysis, data interpretation or content of the manuscript.

### 2.3. Variables

We created two dependent variables:

1. A new purchase of no-alcohol beer: defined at the individual household level as a first purchase of no-alcohol beer that occurred at least 91 days since the household made its first purchase of any form of alcohol, with at least one purchase of regular beer during the 91 days. Households were dichotomized into:
  - Those that had made a new purchase of no-alcohol beer;
  - Those that had never bought no-alcohol beer.

Thus, when comparing the two groups of households, we excluded those that had bought no-alcohol beer but whose purchase did not fit the definition of a new purchase.

2. Volume of no-alcohol beer purchased on day of new purchase of no-alcohol beer and on subsequent purchases, expressed as millilitres.

We used 91 days as the cut-off time based on previous analyses [16].

We created two independent continuous price variables, price of no-alcohol beer and price of regular-strength beer:

1. For households that newly bought no-alcohol beer:
  - No-alcohol beer price: The price per litre of no-alcohol beer paid at the time of the new purchase of no-alcohol beer.
  - Regular-strength beer price: The average price of regular-strength beer per litre paid by the same individual household during the three months prior to the new purchase of the no-alcohol beer.
2. For households that had never purchased no-alcohol beer:
  - No-alcohol beer price: For each unique date a new no-alcohol purchase occurred within a given region or autonomous community, we calculated the average price per litre paid by active, established no-alcohol beer buyers during the preceding three-month window. This average regional price was then assigned to non-purchasing households that had bought any type of alcohol during that same three-month period. If a household matched multiple qualifying windows and was eligible for multiple average prices, the mean of those average prices was applied.
  - Regular-strength beer price: The average price per litre paid by that non-purchasing household during the identical previous three-month window defined in (a). If multiple windows overlapped for a single household, the mean of the corresponding average prices was applied.

Thus, except for the actual price paid when a household newly purchased no-alcohol beer, we used average prices over a three-month period, stratified for the region in which households were located. Ideally, we would have wanted to compare real prices in outlets at the time of purchase for all comparisons, but such data were not available. We chose three-monthly periods to ensure a sufficient number of purchases of no-alcohol beer across the administrative regions of both Great Britain and Spain. We included the calendar time (each sequential three-month period) in our regression models to account for secular trends.

Separately for Great Britain and Spain, we converted the price paid to a percentage scale ranging from 0% (representing the cheapest price in the sample) to 100% (representing the most expensive price in the sample). We then divided this percentage scale by ten, mapping the data onto a standardized 0–10 scale. Consequently, a one-unit change on this transformed scale corresponds to a 10-percentage-point difference relative to the total observed sample price range, rather than a 10% relative change in the absolute retail price per litre. We created seven fixed factors:

1. Two groups of countries, Great Britain and Spain;
2. Based on wealth assets, administrative regions separately in Great Britain and Spain were collapsed into four groups, ranked as wealthy, above average, below average, and poor.
3. Three groups of ages of the main shopper (18–44; 45–64; 65+ years);
4. Four occupation-based social grade groups, ranked as high, above average, below average and low;
5. Three groups of the number of grams of all alcohol regularly purchased (>0–21; >21–70; and >70 g per adult per household per week);
6. Three clustered households of predominantly beer-, wine- and spirit-purchasing households (see [15]). K-means clustering was used to specify the number of three clusters in advance, with clustering based on the proportion of the total grams of alcohol purchased for each household from beer, wines, and spirits;
7. COVID-19 confinement: During 2020, COVID-19 mitigation-measures initiated home confinement and closure of premises such as pubs, bars and restaurants. Since this led to increased purchases of alcohol for home consumption [17], purchase days were grouped into those without confinement and those with confinement.

### 2.4. Statistical Analyses

For the regression analyses, we used SPSS procedure GENLIN [14] in one of two ways:

- When estimating the odds ratios of the association of price with newly buying no-alcohol beer, we used a binomial distribution with a logit link function. We report the association of the fixed factors (household characteristics) with the odds of newly buying no-alcohol beer, compared to a reference group for each category. To determine the extent to which price differences are associated with different odds of newly buying no-alcohol beer by the different elements of each fixed factor (for example, country or age of the main shopper), we repeated the regression analyses including the interaction terms between each of the fixed factors and the two independent price variables. Based on previous publications [8,10,11,15,16,17], the interaction analyses with all seven fixed factors were planned a priori as part of the original study design to comprehensively evaluate how household purchasing behaviours varied by the specified factors. To facilitate interpretation, the adjusted odds ratios (aOR) obtained from the logistic regression models were converted into percentage changes in odds using the following formula: Percentage Change = (aOR − 1) × 100.
- When estimating the association of price with the volume of no-alcohol beer purchased, we used a Gamma distribution with a log link function. The distribution of the continuous volume variable at initial purchase was mildly right-skewed and restricted to positive values (containing no zeros). Accordingly, a Generalized Linear Model with a Gamma distribution and log link was selected to model consumer volume directly on its original scale, thereby avoiding back-transformation bias and preventing distribution over-correction. This structural specification was verified using the model’s Pearson Chi-Square/*df* ratio (0.67) and Deviance/*df* ratio (0.48) and compared against a competing Log-Normal specification using scale-independent metrics: the Gamma GLM achieved a lower Root Mean Squared Error of 1517.75 vs. Log-Normal RMSE of 1570.40, and a higher Efron’s Pseudo-R^2^ of 0.0376 vs. Log-Normal of 0.0302. A plot of Deviance Residuals vs. predicted values for the Gamma GLM demonstrated a stable, symmetric distribution of Deviance residuals centred around zero across the range of predicted values, as shown in Supplementary Figure S1.

We report the same associations as in point (i) above. To facilitate interpretation, we report percentage changes in volume using the following formula: Percentage Change = (EXP(B) − 1) × 100.

### 2.5. Sensitivity Analyses

We undertook the following sensitivity analyses:

- We repeated the main analysis varying the time interval of the definition of a new purchase of no-alcohol beer from 91 days to both 60 days and 121 days.
- Given that the analyzed data set excluded households that had purchased alcohol less than once every three months, we undertook an additional regression analysis of all households to assess if their exclusion would have any mathematical impact on the main model coefficients.
- When estimating the association of price with the volume of no-alcohol beer purchased, we repeated the main analysis using the Log-Normal model.
- Given that the British data (2015–2020) and the Spanish data (2017–2022) cover different time windows, and that the timing and severity of COVID-19 impacts varied between the two countries, we created a new dichotomous variable, time period, The dichotomous variable is either 2017-2020 or all years (2015 to first quarter 2022). In the regression model, we introduced a three-way interaction term, country * time period * price of no-alcohol beer to investigate if there was any overall structural change between the two time periods.

## 3. Results

### 3.1. Data Set and General Characteristics

In Great Britain, the data set analyzed (those households that made at least one purchase of alcohol at least every three months) included 68,712 households (out of 80,667 in the panel), with 4.9 million separate alcohol purchases, 97.5% of all purchases of the full data set. In Spain, the analyzed data set included 16,978 households (out of 18,954 in the panel), with 1.3 million separate alcohol purchases, 97.0% of all purchases of the full data set.

**Figure 1:**
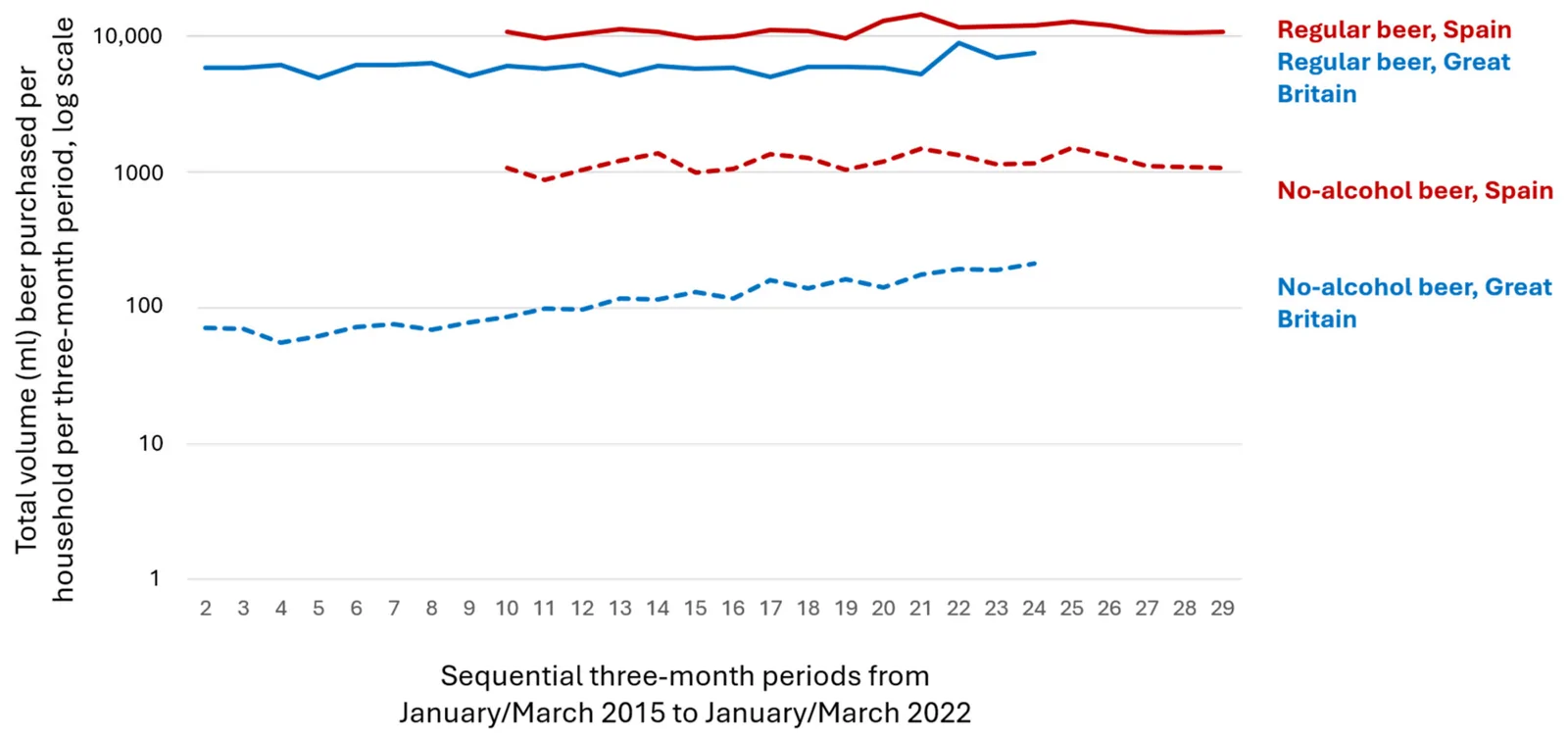
Average volume (mL) of regular (solid line) and no-alcohol (dashed line) beer purchased per household per three-month period from period 1 (January/March 2015) to period 29 (January/March 2022) (NB: log scale).

**Figure 2:**
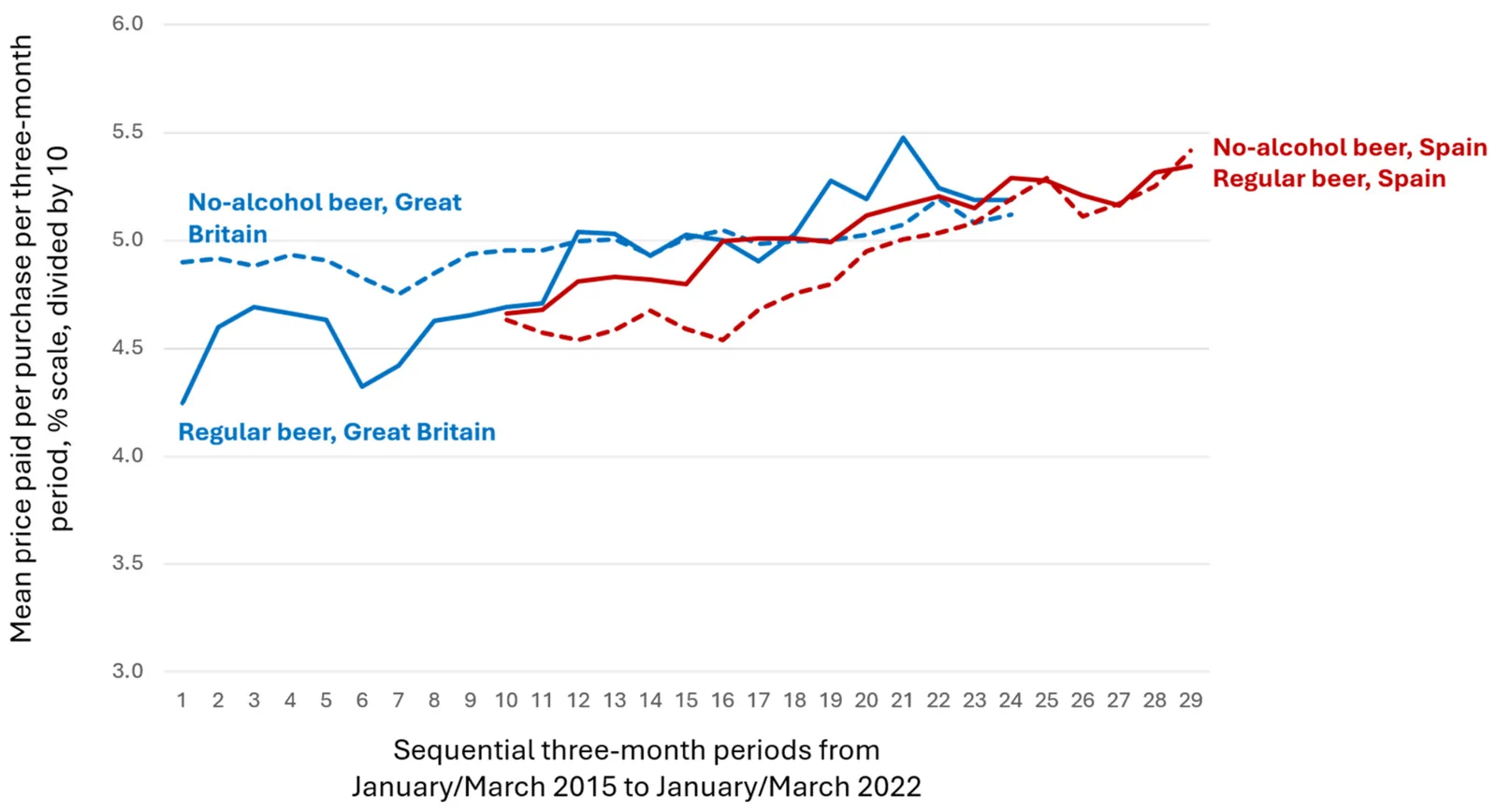
Average price paid per three-month period for one litre of regular (solid line) and no-alcohol (dashed line) beer. (NB: the prices have been scaled to a percentage scale, ranging from 0% (cheapest price) to 100% (most expensive price), divided by 10.

In Great Britain, the volume of purchases of no-alcohol beer increased gradually over time, whereas the volume remained stable in Spain, as shown in Figure 1 (log scale). In both countries, the prices of beers increased over time (Figure 2).

### 3.2. Associations of Price with Newly Purchasing No-Alcohol Beer

Of the 85,690 households in both countries, 7509 (8.8%) newly bought no-alcohol beer (5.2% in Great Britain, and 23.1% in Spain). For every 10-percentage-point decrease in price relative to the sample price range for no-alcohol beer, there was a 157% (95% CI = 148% to 167%) increase in the odds of households newly buying no-alcohol beer (Table 1, middle column), representing an absolute increase of 11.1 percentage points (from 8.8% to 19.9%). Conversely, for every 10-percentage-point increase in price relative to the sample range for regular-strength beer, there was an 11.3% (95% CI = 10.0% to 12.7%) increase in the odds of households newly buying no-alcohol beer, as shown in Table 1, middle column, representing an absolute increase of 0.9 percentage points (from 8.8% to 9.7%).

For sensitivity analysis (i), when defining a new purchase of a no-alcohol beer as a purchase of no-alcohol beer that occurred at least 60 days (rather than the 91 days) since the household made its first purchase of any form of alcohol, there was a 163% (95% CI = 154% to 172%) increase in the odds for every 10-percentage-point decrease in price relative to the sample price range for no-alcohol beer and 11.4% increase (95% CI = 10.1% to 12.8%) for every 10-percentage-point increase in price relative to the sample range for regular-strength beer. The respective associations when the time interval was 121 days were 160% (95% CI = 150% to 170%) and 11.5% (95% CI = 10.1% to 12.9%).

For sensitivity analysis (ii), we added the 13 households from the excluded sample (those purchasing less than once every three months) that fit the criteria of newly purchasing no-alcohol beer to the 7509 of the main sample, and found no difference in the results: For every 10-percentage-point decrease in price relative to the sample price range for no-alcohol beer, there was a 155% (95% CI = 146% to 164%) increase in the odds of households newly buying no-alcohol beer, and for every 10-percentage-point increase in price relative to the sample range for regular-strength beer, there was an 11.0% (95% CI = 9.8% to 12.2%) increase in the odds of households newly buying no-alcohol beer.

The percentage change in odds of newly buying no-alcohol beer was lower for British households than for Spanish households; was higher for more wealthy regions than for poorer regions; did not differ consistently by age; was higher for those with higher social grade than those with lower social grade; was higher for those that tended to buy less grams of alcohol overall; was higher for households that predominantly bought wine as opposed to beer; and did not differ by confinement status, as shown in Table 1, middle column.

For the 7509 households that newly bought no-alcohol beer, for every 10-percentage-point decrease in price relative to the sample price range for no-alcohol beer, the volume of no-alcohol beer purchased increased by 8.4% (95% CI = 7.3% to 9.6%), as shown in Table 1, right-hand column. A relatively higher price of regular-strength beer was not significantly associated with the volume of purchased no-alcohol beer.

For sensitivity analysis (iii), when using the Log-Normal model, the estimate was very similar: for every 10-percentage-point decrease in price relative to the sample price range for no-alcohol beer, the volume of no-alcohol beer purchased increased by 8.7% (95% CI = 7.6% to 9.8%); a relatively higher price of regular-strength beer was not significantly associated with the volume of purchased no-alcohol beer.

The household characteristics had little relationship with the volume of no-alcohol beer purchased, except for British households which purchased less volume than Spanish households; households with higher social grade that purchased a greater volume than households with lower social grade; and, during confinement, when a greater volume was purchased, as shown in Table 1, right-hand column.

### 3.3. Differential Associations of Price by Household Characteristics

#### 3.3.1. With Respect to the Percentage Change in Odds of Newly Buying No-Alcohol Beer

A lower price of no-alcohol beer had a larger association in Great Britain than in Spain. For every 10-percentage-point decrease in price relative to the sample price range for no-alcohol beer, the percentage change in odds of buying no-alcohol beer was 206% (95% CI = 184% to 230%) greater in Great Britain than in Spain (see Supplementary Table S1). Whereas a difference in the price of regular-strength beer had a slightly greater association with the likelihood of newly buying no-alcohol beer in Spain than in Great Britain. For every 10-percentage-point increase in price relative to the sample range for regular-strength beer, the percentage change in odds of buying no-alcohol beer was 5.5% (95%CI = 3.2% to 7.8%) greater in Spain than in Great Britain.

For sensitivity analysis (iv), the omnibus Type III Wald test found no overall structural change over time (*p* = 0.952): for every 10-percentage-point decrease in price relative to the sample price range for no-alcohol beer, the percentage change in odds of buying no-alcohol beer in Spain for the years 2017 to 2020 compared to all years were −0.2% (95%CI= −6.2% to 6.2%), and for Great Britain were 0.2% (95%CI = −9.4% to 10.8%). In other words, the price associations observed in Great Britain relative to Spain remained the same during the 2017–2020 window as for the whole of the sample years.

By other household characteristics, for every 10-percentage-point decrease in price relative to the sample price range for no-alcohol beer, the percentage change in odds of newly buying no-alcohol beer was 24.7% (95%CI = 17.7% to 31.1%) greater for households that regularly bought more than 70 g of alcohol/week compared with those that bought 21 or less grams per week; and, for every 10-percentage-point decrease in price relative to the sample price range for no-alcohol beer, the percentage change in the odds of newly buying no-alcohol beer was 8.7% (95%CI 2.9% to 14.9%) higher during the days of COVID confinement compared with that during no confinement. For higher prices of regular beer, there were no clear differential associations by household characteristics (see Supplementary Table S1).

#### 3.3.2. With Respect to the Volume of No-Alcohol Beer Purchased at the Time of Newly Buying No-Alcohol Beer

A lower price of no-alcohol beer had a slightly greater association in Great Britain than in Spain (see Supplementary Table S2). For every 10-percentage-point decrease in price relative to the sample price range for no-alcohol beer, the purchased volume of no-alcohol beer was 7.5% (95% CI 4.4% to 10.8%) greater in Great Britain than in Spain. Otherwise, differences in the prices of no-alcohol beer and regular-strength beer had no clear differential associations with the volume of no-alcohol beer purchased at the time of newly buying no-alcohol beer by the different household characteristics (see Supplementary Table S2).

### 3.4. Subsequent Purchases

Of the 7509 households that made a new purchase of no-alcohol beer, 3660 remained in the panel for at least an additional three months. Among this tracked subset, 2160 households (59.0%) went on to make at least one further purchase of no-alcohol beer. During the subsequent purchases, for every 10-percentage-point decrease in price relative to the sample price range of no-alcohol beer, a 9.6% greater volume of no-alcohol beer was purchased (95% CI = 8.5% to 10.7%).

## 4. Discussion

The analyses found that a 10-percentage-point decrease in price relative to the sample price range of no-alcohol beer was associated with a 157% increase in the odds of a new purchase of no-alcohol beer (representing an absolute increase of 11.1 percentage points (from 8.8% to 19.9%) and an 8.4% increase in the volume bought. For regular-strength beer, higher prices relative to the sample range were associated with an increased odds of households beginning to buy no-alcohol beer, but not with the volume bought, suggesting that, with respect to the price of regular beer, the potential impact occurs primarily in terms of whether households choose to purchase no-alcohol beer for the first time, rather than the volume purchased.

We restricted the analyses to the 86% of households in the panels that made at least one purchase of any alcohol product at least every three months, such households purchasing just under 98% of all beer purchased by the full panels. Adding the excluded households (those that made an alcohol purchase less than once every three months) to the analysis made no difference to the results. We defined a new purchase of no-alcohol beer as that which occurred at least 91 days since a household made its first purchase of any form of alcohol. Varying this time to either 60 days or 121 days made little difference to the main results.

Associated price effects of no-alcohol beer with a new purchase of no-alcohol beer differed across the two studied markets, being larger in Great Britain (an emerging market for no-alcohol beer) than in Spain (a more mature market).

Evidence on the impact of price on purchasing of no- and low-alcohol products remains limited, with most existing studies focusing on wider alcohol pricing policies rather than directly on no-alcohol beer [4,18]. Research on minimum unit pricing in Scotland and Wales, for example, shows that pricing policies can shift purchases from higher- to lower-strength beers, leading to reductions in overall alcohol consumption [12,19].

Although, as with alcohol, available evidence is very limited, similar patterns have been observed in the food literature, where studies suggest that higher prices can limit adoption of plant-based meat alternatives, such as burgers, while lower prices may encourage more consumers to try and adopt alternative healthier burgers [20,21]. Although the products differ (beer and burgers), this evidence shows the important role of price in encouraging consumers to substitute toward healthier options.

As reported elsewhere [22], the present analysis found that newly purchasing no-alcohol beer tended to be a higher socio-economic phenomenon: it is important to ensure that increased uptake of no-alcohol beer, and no- and low-alcohol products more generally, do not exacerbate health inequalities [8].

A strength of the study is that we obtained product bar code data from many analyzed households with many separate alcohol purchases over five to six years. Moreover, the inclusion of two countries with different alcohol markets and cultures [2] enhances the generalizability of the findings outside just one country.

A number of limitations are acknowledged. First, except for the actual price paid when a household newly purchased no-alcohol beer, we had to rely on average prices over a three-month period, stratified for the region in which households were located. Ideally, we would have wanted to compare real prices in outlets at the time of purchase for all comparisons, but we were not able to do so as the Kantar panel provided household purchase data and not store data. Assigning regional three-month averages to never-purchasing households as a proxy for the prices they faced introduces potential measurement errors: as new purchasers are evaluated using actual prices while never-purchasers are evaluated using aggregate averages, the price variables for the two groups are constructed asymmetrically, which could systematically bias the estimated price associations, potentially overestimating the consumer price sensitivity.

Second, the actual price paid often reflects promotions, product attributes, and retail availability. If these factors independently drive no-alcohol beer purchases, our coefficients may capture these unobserved elements, introducing omitted variable bias. Furthermore, for first-time buyers, both unit price and total volume are derived from the exact same transaction. This mathematical dependency introduces structural confounding. Consequently, the observed association between lower unit prices and higher volumes may partially reflect a structural artefact—such as consumers buying larger pre-packaged quantities or leveraging volume discounts—rather than a behavioural shift driven purely by a lower base price. Here, it is important to note that a previous study using British data, predominantly for the years 2015–2018, studying the potential impact of prices and price promotions on purchases of beer [12], found for no-alcohol beer similar volume increases from lower prices regardless of whether the no-alcohol beer was on price promotion.

Third, several structural differences between the household panels warrant caution: the datasets span different time horizons (2015–2020 for Great Britain and 2017–2022 for Spain), thus capturing distinct waves, macro-economic contexts, and policy responses to the COVID-19 pandemic; and the two countries represent different stages of market development. Although our sensitivity analysis found no difference in results when analyzing contemporary time periods in both countries (2017 to 2020), future research utilizing harmonized market stages could help isolate purely geographic differences in consumer behaviour.

Fourth, our findings regarding the long-term adoption of no-alcohol beer must be interpreted with caution due to limited follow-up coverage. While a majority of the retained subset (59.0%) made a subsequent purchase within three months, more than half of the initial new-purchaser cohort could not be tracked long-term due to panel attrition. Consequently, true behavioural persistence across the entire sample remains unobserved, and further longitudinal research with extended retention windows is required to confirm permanent substitution patterns. Here, it is important to note that a previous analysis of British household purchase data reported substitution with households newly purchasing no-alcohol beer subsequently reducing purchases of regular-strength beer that was maintained over two-years of follow-up [10].

Fifth, the analysis is conducted at the household level and cannot identify which members consumed the products, nor whether alcohol was shared equally within households.

Sixth, the study captures purchases rather than actual consumption and does not consider alcohol consumed outside the home.

## 5. Conclusions and Policy Implications

Alcohol consumption is toxic to human health across a wide range of health outcomes [23,24], with increased risk occurring at approximately one drink a day [25], and, when methodological study characteristics are taken into account, there appears no lasting evidence for a protective effect at small doses [26]. Reducing alcohol consumption can result in a wide range of improved health outcomes [27]. Previous analyzed data from both Great Britain and Spain suggested that households that newly purchased no-alcohol beer subsequently reduced purchases of regular-strength beer [10,15], leading to less purchases of grams of alcohol overall, and thus, potentially, lower levels of alcohol consumption and health improvement.

The results of the present study suggest that price differences between no-alcohol beer and regular-strength beer are associated with potential increases in purchases of no-alcohol beer. While the reported estimates in the present analysis are associative rather than causal, they provide a possible benchmark illustrating how consumer purchasing patterns might align with coordinated pricing policies.

To explore the potential for public health gains from no- and low-alcohol beers, future policy frameworks could consider introducing pricing policies that lower the prices of no and low-alcohol beers while increasing those of regular-strength beers. Specifically, alcohol taxes could be set with a fixed duty per gram of alcohol (zero for no-alcohol beers), with the fixed duty rates per gram increasing with increasing alcohol by volume of the product. Such a tax system would result in higher-strength products being taxed at higher rates, potentially encouraging producers to lower the alcoholic strength of their products [8]. Whilst our findings are associational, such policies may support consumer changes toward lower-alcohol alternatives, contributing to reductions in alcohol consumption at the population level [6]. To optimize potential impact, pricing measures should be supported by wider alcohol policies that improve availability of no- and low-alcohol products, clear labelling, and responsible marketing, so that all groups can benefit from moving to lower-alcohol options [8]. As with all pricing policies, care should be taken in avoiding relevant potential unintended consequences, such as substitution toward other alcohol products and the regressivity of taxation for low-income groups [8].

## Figures and Tables

**Table 1:** Associations between both prices and household characteristics and: (i) percentage change in the odds of a household newly buying versus never buying no-alcohol beer; and (ii) percentage change in volume of no-alcohol beer purchased at time of new purchase of no-alcohol beer.

Independent (Predictor) Variables		Percentage Change in Odds (95% Confidence Interval) of Households Newly Buying No-Alcohol Beer (*n* 7509) Compared to Households Never Buying No-Alcohol Beer (*n* 78,181) ^1^	Percentage Change in Volume (95% Confidence Interval) of No-Alcohol Beer Purchased at Time of New Purchase of No-Alcohol Beer (*n* 7509) ^2^
Price	For every 10% point decrease in price of no-alcohol beer relative to sample price range	156.9 (147.7 to 166.5)	8.4 (7.3 to 9.6)
	For every 10% point increase in price of regular beer relative to sample price range	11.3 (10.0 to 12.7)	−1.5 (−3.5 to 0.5)
Factors			
Country	Great Britain	−69.6 (−72.4 to −66.5)	−24.1 (−28.7 to −19.2)
	Spain	Reference Group	
Wealth of Region	Wealthy	48.6 (38.8 to 59.0)	−7.9 (−11.8 to −3.9)
	Above average	13.4 (5.3 to 22.1)	−1.3 (−6.0 to 3.6)
	Below average	−9.4 (−15.8 to −2.6)	−6.5 (−10.8 to −1.9)
	Poor	Reference Group	
Age	18–44	3.4 (−4.0 to 11.4)	−2.0 (−6.8 to 3.0)
	45–64	−13.3 (−19.3 to −6.8)	0.4 (−4.2 to 5.4)
	65+	Reference Group	
Social Grade	High	9.3 (0.5 to 18.9)	12.2 (6.0 to 18.7)
	Above average	12.8 (4.6 to 21.7)	11.7 (6.2 to 17.4)
	Below average	2.5 (−5.7 to 11.4)	6.2 (0.4 to 12.2)
	Low	Reference Group	
Drinking Group	0–21	239.7 (218.2 to 262.6)	−8.1 (−12.0 to −4.1)
	>21–70	78.4 (67.2 to 90.3)	−2.4 (−6.5 to 1.9)
	>70	Reference Group	
Predominant Beverage	Wine	7.2 (1.2 to 13.5)	−3.9 (−7.4 to −0.2)
	Spirits	6.3 (−0.6 to 13.6)	5.4 (0.9 to 10.1)
	Beer	Reference Group	
COVID-19 Confinement	Confinement	−4.5 (−12.9 to 4.6)	8.7 (2.2 to 15.6)
	No confinement	Reference Group	

^1^ The regression model includes Country, Wealth of Region, Age, Social grade, Drinking group, Predominant beverage, COVID-19 confinement, and Calendar Time (each sequential three-month period) as fixed factors and the price of regular beer and the price of no-alcohol beer as covariates, with a binomial distribution and logit link function. ^2^ The regression model includes Country, Wealth of Region, Age, Social grade, Drinking group, Predominant beverage, COVID-19 confinement and Calendar Time (each sequential three-month period) as fixed factors and the price of regular beer and the price of no-alcohol beer as covariates, with a Gamma distribution with a log link function.

## Data Availability

No additional data available. Kantar Worldpanel data cannot be shared due to licencing restrictions.

## Supplementary Materials

The following supporting information can be downloaded at: https://www.mdpi.com/article/10.3390/nu18203310/s1. Figure S1. Deviance Residuals vs. Predicted Values for the Primary Gamma GLM. Table S1. Logistic regression analysis of household characteristic–price interactions on the odds (expressed as percentage change) of households transitioning to purchasing no-alcohol beer. Table S2. Regression analysis of household characteristic–price interactions on the percentage change in the volume of no-alcohol beer purchased at the time of initial purchase.
