# Supplementary material for: The Association Between Price and Household Purchasing of No-Alcohol Beer in Great Britain and Spain
Source: Nutrients. 2026 Oct 9;18(20):3310. doi: 10.3390/nu18203310 (PMC13708731; doi:10.3390/nu18203310)
Supplement: Supplementary file 1 [file nutrients-18-03310-s001.zip › nutrients-4574438-supplementary.pdf]

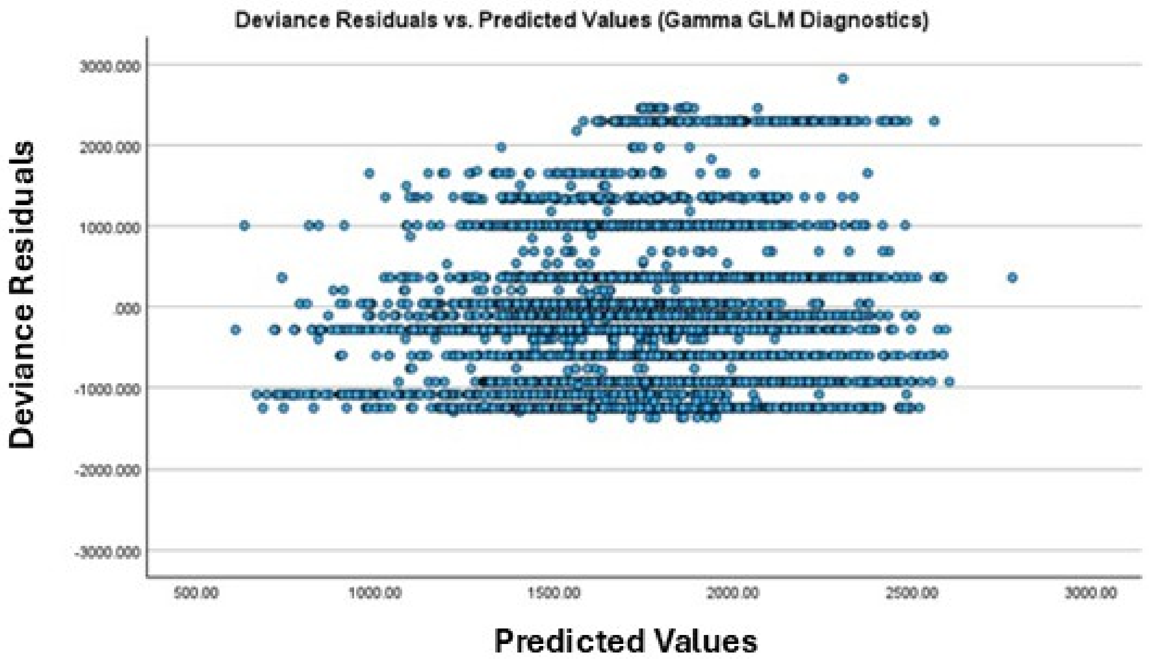

Supplementary Figure S1. Deviance Residuals vs. Predicted Values for the Primary Gamma GLM.

Supplementary Table S1. Logistic regression analysis of household characteristic–price interactions on the odds (expressed as percentage change) of households transitioning to purchasing no-alcohol beer."

Fixed factors		Price of no-alcohol beer: Percentage change in odds (95% confidence interval) of households newly buying no-alcohol beer (n 7,509) compared to households never buying no-alcohol beer (n 78,181)[1]	Price regular beer: Percentage change in odds (95% confidence interval) of households newly buying no-alcohol beer (n 7,509) compared to households never buying no-alcohol beer (n 78,181)[1]
Country	Great Britain	206.3 (184.2 to 230.2)	-5.5 (-7.8 to -3.2)
	Spain	Reference group	
Wealth of Region	Wealthy	15.3 (6.0 to 25.4)	-0.8 (-3.9 to 2.4)
	Above average	-37.5 (-42.9 to -31.5)	-2.3 (-5.8 to 1.2)
	Below average	1.5 (-7.6 to 11.5)	-1.1 (-4.5 to 2.4)
	Poor	Reference group	
Age	18-44	0.0 (-1.4 to 1.3)	0.3 (-0.8 to 1.4)
	45-64	-0.1 (-1.3 to 1.1)	0.1 (-0.8 to 1.0)
	65+	Reference group	
Social grade	High	-0.1 (-1.5 to 1.4)	-0.1 (-0.9 to 0.7)
	Above average	-0.1 (-1.3 to 1.2)	-0.1 (-0.8 to 0.6)
	Below average	-0.1 (-1.6 to 1.4)	0.0 (-0.8 to 0.8)
	Low	Reference group	
Drinking Group	0-21	-24.7 (-31.1 to -17.7)	-1.5 (-4.6 to 1.7)
	>21-70	-8.9 (-16.7 to -0.4)	-0.9 (-4.0 to 2.3)
	>70	Reference group	
Predominant beverage	Wine	0.0 (-1.1 to 1.1)	0.0 (-0.6 to 0.6)
	Spirits	0.0 (-1.2 to 1.1)	0.0 (-0.7 to 0.7)
	Beer	Reference group	
Confinement status	Confine	8.7 (2.9 to 14.9)	4.0 (-3.2 to 11.7)
	No confine	Reference group	

[1]Separate regression models were run for each of the household groupings (Wealth of Region, Age, Social grade, Drinking Group, Predominant beverage, and Confinement status), with for each model country and Calendar Time (each sequential three-month period) included as a fixed factors, the price of regular beer and the price of no-alcohol beer as covariates, and the interaction term of the relevant household grouping (e.g., Wealth of Region*price of regular beer, Wealth of Region*price of no-alcohol beer), with a binomial distribution and logit link function.

Supplementary Table S2. Regression analysis of household characteristic–price interactions on the percentage change in the volume of no-alcohol beer purchased at the time of initial purchase.

Fixed factors		Price no-alcohol beer: Percentage change in volume (95% confidence interval) of no-alcohol beer purchased at time of new purchase of no-alcohol beer (n 7,509).[1]	Price regular beer: Percentage change in volume (95% confidence interval) of no-alcohol beer purchased at time of new purchase of no-alcohol beer (n 7,509).[1]
Country	Great Britain	7.5 (4.4 to 10.8)	1.2 (-1.5 to 4.0)
	Spain	Reference group	
Wealth of Region	Wealthy	-0.3 (-4.0 to 3.5)	-3.2 (-6.6 to .4)
	Above average	-2.1 (-6.1 to 2.1)	-0.9 (-4.9 to 3.3)
	Below average	-.2 (-4.3 to 4.1)	0.8 (-3.2 to 5.0)
	Poor	Reference group	
Age	18-44	-0.4 (-4.5 to 3.9)	1.5 (-2.5 to 5.7)
	45-64	-1.1 (-5.2 to 3.1)	-0.1 (-4.1 to 4.0)
	65+	Reference group	
Social grade	High	1.1 (-3.5 to 5.9)	2.1 (-2.4 to 6.8)
	Above average	-1.5 (-5.6 to 2.9)	2.1 (-2.0 to 6.3)
	Below average	-0.7 (-5.4 to 4.4)	-1.9 (-6.3 to 2.7)
	Low	Reference group	
Drinking Group	0-21	0.1 (-3.7 to 3.9)	-1.1 (-4.6 to 2.6)
	>21-70	1.4 (-2.4 to 5.2)	-0.4 (-3.9 to 3.3)
	>70	Reference group	
Predominant beverage	Wine	3.3 (-0.1 to 6.8)	2.1 (-1.1 to 5.3)
	Spirits	5.0 (1.0 to 9.1)	2.9 (-0.7 to 6.6)
	Beer	Reference group	
Confinement status	Confine	3.0 (-1.5 to 7.7)	5.2 (-2.3 to 13.1)
	No confine	Reference group	

[1]Separate regression models were run for each of the household groupings (Wealth of Region, Age, Social grade, Drinking Group, Predominant beverage, and Confinement status), with for each model country and Calendar Time (each sequential three-month period) included as fixed factors, the price of regular beer and the price of no-alcohol beer as covariates, and the interaction term of the relevant household grouping (e.g., Wealth of Region*price of regular beer, Wealth of Region*price of no-alcohol beer), with a gamma distribution with a log link function.
